# In Situ Imaging of O-Linked β-N-Acetylglucosamine Using On-Tissue Hydrolysis and MALDI Mass Spectrometry

**DOI:** 10.3390/cancers15041224

**Published:** 2023-02-15

**Authors:** Edwin E. Escobar, Erin H. Seeley, Jesús E. Serrano-Negrón, David J. Vocadlo, Jennifer S. Brodbelt

**Affiliations:** 1 Department of Chemistry, The University of Texas at Austin, Austin, TX 78712, USA; 2 Department of Molecular Biology and Biochemistry, Burnaby, BC V5A 1S6, Canada; 3 Department of Chemistry, Simon Fraser University, Burnaby, BC V5A 1S6, Canada

**Keywords:** mass spectrometry imaging, MALDI, ultraviolet photodissociation, O-glycosylation, in situ digestion, biomarkers, O-GlcNAc

## Abstract

**Simple Summary:**

Post-translational O-glycosylation of proteins with N-acetylglucosamine serves as a cellular regulator that has been linked to cancer. This sugar’s variability is emerging as a metabolic biomarker in cancer, and here we investigate the use of mass spectrometry imaging to visualize the location of this sugar in primary tumor sections. O-GlcNAc hydrolase, an enzyme specific to N-acetylglucosamine, has been employed for on-tissue hydrolysis of this sugar, providing additional evidence implicating it in tumor growth.

**Abstract:**

Post-translational O-glycosylation of proteins via the addition of N-acetylglucosamine (O-GlcNAc) is a regulator of many aspects of cellular physiology. Processes driven by perturbed dynamics of O-GlcNAcylation modification have been implicated in cancer development. Variability in O-GlcNAcylation is emerging as a metabolic biomarker of many cancers. Here, we evaluate the use of MALDI-mass spectrometry imaging (MSI) to visualize the location of O-GlcNAcylated proteins in tissue sections by mapping GlcNAc that has been released by the enzymatic hydrolysis of glycoproteins using an O-GlcNAc hydrolase. We use this strategy to monitor O-GlcNAc within hepatic VX2 tumor tissue. We show that increased O-GlcNAc is found within both viable tumor and tumor margin regions, implicating GlcNAc in tumor progression.

## 1. Introduction

Aberrant glycosylation patterns are a non-genetically coded process that are a widely documented hallmark of malignant tumor transformation [1,2,3]. Glycans can be covalently N- or O-linked, modifying asparagine or serine and threonine residues, respectively. A few naturally occurring sugars can be combined to create many unique glycan structures in a dysregulated disease state system. For example, atypical glycan branching, upregulated fucosylation, or sialylation of N-glycans have been linked to several cancerous processes [4,5,6]. Reportedly, the effects of altered glycosylation patterns can ultimately allow tumor cells to evade the immune system and increase invasion, causing metastasis [7,8]. N-linked glycosylation as a post-translational modification has, in particular, been extensively studied in its context to cancer and other diseases through traditional proteomics and mass spectrometry imaging [9,10]. The attachment of O-linked β-N-acetylglucosamine (O-GlcNAc) has, by comparison, been much less explored using these approaches. O-GlcNAc is distinct from other common forms of protein glycosylation in that it occurs both post- and co-translationally within the nucleus and cytoplasm of cells [11].

**Figure 1:**
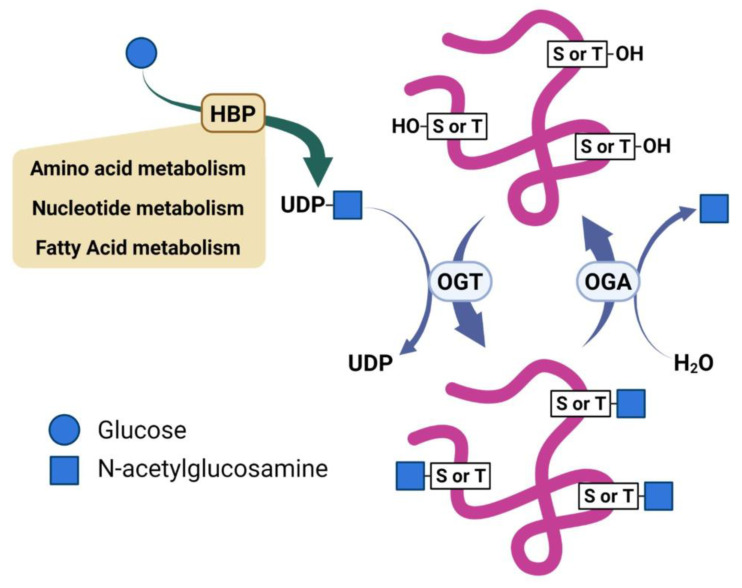
Scheme illustrating regulation of O-GlcNAc by nutrients and reciprocal activities of O-GlcNAc transferase (OGT) and O-GlcNAc hydrolase (OGA).

Two enzymes mediate the enzymatic cycling of O-GlcNAc; O-GlcNAc transferase (OGT) installs the sugar on target proteins, and O-GlcNAc hydrolase (OGA) removes this modification [12]. Uridine diphosphate (UDP)-GlcNAc, a product of the Hexosamine Biosynthetic Pathway (HBP), serves as the donor of the β-N-acetylglucosamine residue used by OGT to install the O-GlcNAc modification on serine or threonine residues of target proteins (Figure 1) [13]. The HBP is recognized as a minor pathway involved in glucose metabolism. However, reports describe the HBP as a metabolic processor, which serves to integrate inputs from glutamate, fatty acid, and nucleotide metabolism, as well as oxidative phosphorylation [14,15]. As such, the reversible nature of O-GlcNAcylation enables it to serve as a sensor and regulator of diverse cellular processes. Dysregulation of O-GlcNAcylation is recognized as a potential contributor to several diseases, such as cancer and neurodegeneration [16,17]. Despite its importance, the cellular and molecular mechanisms underlying how O-GlcNAcylation regulates these processes remain elusive. Emerging data show that O-GlcNAc contributes to regulating various signaling pathways through both transcriptional and post-transcriptional mechanisms [18]. Several of these signal pathways are notable oncogenic pathways that have already been implicated in cancers [19,20,21]. Thus, O-GlcNAcylation is directly involved in producing downstream effects on gene expression, metabolism homeostasis, protein translation, and protein–protein interactions dysregulated in cancer [15,22]. Proteomic studies have found over a thousand proteins modified with O-GlcNAc lending the potential for this modification to contributes through diverse mechanisms to various biological effects [11,23,24,25].

Understanding the cellular dynamics of the O-GlcNAc modification is critical for elucidating its functional roles in both physiological and diseased states as the roles of this modification in various diseases is deciphered. Accordingly, mapping protein O-GlcNAcylation has recently become a topic of interest. However, accurate and precise MS-based mapping of O-GlcNAc has remained an obstacle because the stoichiometric abundances of O-GlcNAc present on target proteins are often low, and the high lability of the glycosidic linkage of O-GlcNAc residues results in the loss of the sugar residue for some mass spectrometric (MS) approaches. A few methods exist for in situ imaging of O-GlcNAc. Levels are typically monitored by immunoblotting with a general O-GlcNAc antibody, specific to a limited set of O-GlcNAc-modified proteins. Although effective, this strategy affords little opportunity to identify novel proteins undergoing changes in glycosylation. Chemically-specific imaging methods have evolved to target O-GlcNAcylation via fluorescence-based techniques, such as fluorescence lifetime imaging microscopy coupled to Förster resonance energy transfer [26,27], azide-alkyne click-chemistry [28,29], and metabolic fluorescent labeling approaches using the addition of exogenous recombinant glycosyltransferases [30,31,32].

An alternative approach for in situ analysis, matrix-assisted laser desorption ionization (MALDI) mass spectrometry imaging (MSI), has emerged, which allows the determination of the spatial distribution of endogenous metabolites, peptides, or proteins [33,34]. In recent MSI-based studies focusing on N-glycosylation, thin tissue slices were washed and incubated with de-N-glycanase (PNGase F) to release the N-linked glycans prior to MALDI analysis [35,36,37,38]. This MALDI MSI strategy revealed marked nuance in changes in N-glycans within benign tissues versus regions of tumors [9,39,40,41,42,43,44,45,46]. Furthermore, multiple groups have also demonstrated the in situ derivatization of glycans and sequential enzymatic glycan digestion procedures prior to MSI to enhance the determination of isomeric glycan linkages or increase detection sensitivity, ultimately expanding the information that can be obtained [47,48,49,50,51,52,53].

Spatial mapping of the distributions of glycans in tissues can be augmented by detailed structural characterization via tandem mass spectrometry (MS/MS), the premier strategy for characterization of peptide sequences and localization of modifications [54,55]. A variety of MS/MS methods, including higher-energy collision dissociation (HCD), electron transfer dissociation (ETD), hybrid methods such as the combination of ETD with supplementary collisional energy (EThcD), and photon-based ultraviolet dissociation (UVPD), have proven successful. Each MS/MS method provides amino acid and glycan sequence information; however, EThcD and UVPD stand out by providing complete glycopeptide characterization [56,57,58].

Here, we describe the use of a GH84 OGA homologue for on-tissue release of GlcNAc for MS-based MALDI imaging. We combined on-tissue hydrolysis and MALDI imaging along with in situ peptide extractions prior to LC-UVPD-MS/MS to characterize O-GlcNAc protein modifications in rabbit tumor tissue. We evaluated the specificity gained by combining spatial imaging and proteomics, particularly focusing on the use of MALDI-MSI to visualize GlcNAc after sequential on-tissue enzymatic reactions to remove interfering N-glycans and enzymatically release GlcNAc. 

## 2. Results

In this work we aimed to use MALDI MS imaging to monitor GlcNAc that was enzymatically released from modified serine and threonine residues of glycoproteins, along with bottom-up proteomics to characterize the glycopeptides to allow inference of the original glycoproteins.

**Figure 2:**
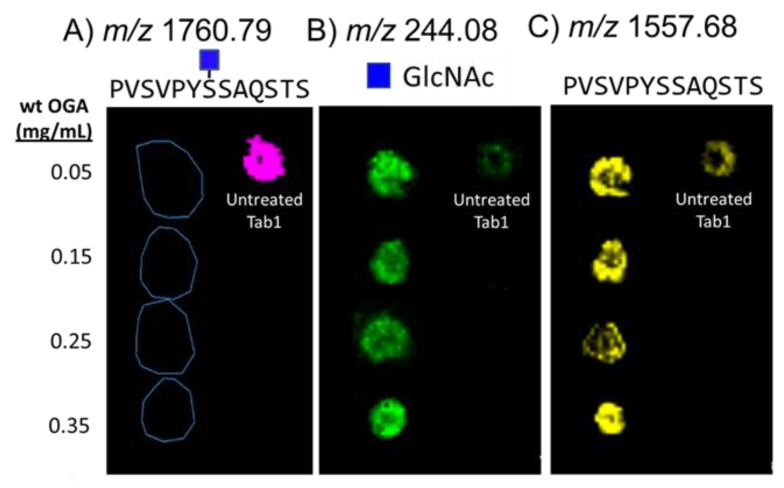
MALDI-MS maps showing the composition of five spots after deposition of O-GlcNAc-modified TAB1 glycopeptide on a slide and treatment with OGA. The extracted ion signals correspond to; (**A**) sodium-cationized glycopeptide (*m*/*z* 1760.79), (**B**) sodium-cationized GlcNAc (*m*/*z* 244.08), and (**C**) sodium-cationized non-glycosylated peptide (*m*/*z* 1557.68). The spot on the right side of each panel is a spot of the glycopeptide that had not been treated with OGA. The four spots on the left side of each panel indicate OGA treatment using four different concentrations of OGA, followed by incubation for two hours.

As a first step, we evaluated the efficacy of on-slide hydrolysis of O-GlcNAc with Bacteroides thetaiotamicron β-N-acetylglucosaminidase (BtOGA), a bacterial OGA homologue from glycoside hydrolase family GH84 that cleaves O-GlcNAc [59] using a standard glycopeptide, O-GlcNAc-modified TAB1^389−401^ (biotin-PVSVPYS395SAQSTS) [58,60]. To optimize enzymatic reaction conditions, we sprayed BtOGA onto dried spots of the TAB1 glycopeptide on glass slides. After incubation and matrix application, we analyzed the glycopeptide spots with MALDI-MS to monitor the extracted ion images for the key products, (TAB1 glycopeptide (*m*/*z* 1760.69); GlcNAc (*m*/*z* 244.08), and deglycosylated TAB1 (*m*/*z* 1557.68), each as sodium-cationized species (Figure 2). The untreated spot showed a dominant signal for the TAB1 glycopeptide (Figure 2A) and very low abundances of GlcNAc (Figure 2B) and unglycosylated TAB1 (Figure 2C). We attributed the detection of unglycosylated peptide to the intrinsic lability of the β-O-glycosidic linkage of GlcNAc residue to the glycopeptide [12], which leads to deglycosylation (Figure S1). Four different concentrations of BtOGA (0.05–0.35 mg/mL) were applied to the dried TAB1 glycopeptide spots and reacted for two hours at 30 °C in a humidity-controlled chamber. These enzyme treatments successfully cleaved O-GlcNAc from the glycopeptide as evidenced by the notable abundances of GlcNAc and deglycosylated peptide ions (Figure 2B,C) along with the disappearance of the glycosylated peptide (Figure 2A). As expected, treatment with 0.35 mg/mL gave the most abundant signals for the released GlcNAc, and we adopted this BtOGA concentration for downstream on-tissue reactions. An analysis of the spots corresponding to the treatment with 0.25 mg/mL OGA revealed comparatively lower abundances of both GlcNAc and the non-glycosylated peptide. Although the glycopeptide and subsequent enzyme treatment were carefully controlled to minimize variations in adherence and evaporative effects on glass slides, some “coffee-ring” effects were observed, and we speculated that this accounts for some of the variations seen in the spots.

To determine a lower limit of detection (LOD) for GlcNAc, we spotted a serial dilution of GlcNAc onto Carnoy’s fluid-washed mouse liver tissue sections. A detailed account of the LOD calculation is included in the experimental section. Briefly, the on-tissue limit of detection was determined based on the quantity of GlcNAc deposited per area (in mass) of sectioned tissue. Given a MALDI resolution of 100 µm, each spot was estimated as a circle with a diameter of 15 pixels (1.5 mm). Using a tissue density of 1.05 g/cm^3^, each spot was determined to average 22.3 µg of the tissue mass. The ion of *m*/*z* 226.07, corresponding to the sodium-cationized water-loss oxonium ion, was detected with regions to which the GlcNAc solution was applied (Figure S2). This corresponded to an absolute detection limit of 37 ng GlcNAc per gram tissue (based on the average width measured from the laser raster pattern (Figure S3). There was a marked difference in sensitivity limitations observed when tracking sodium-cationized GlcNAc (*m*/*z* 244.08) (Figure S2C) or the water-loss GlcNAc oxonium ion (*m*/*z* 204.08) (Figure S2D). The best sensitivity was observed when tracking the sodium-cationized water-loss GlcNAc oxonium ion (*m*/*z* 226.07) (Figure S2E). Thus, to maximize sensitivity and optimize the detection of GlcNAc in tissue, the sodium-cationized water-loss GlcNAc oxonium ion (*m*/*z* 226.07) was used as the marker ion to monitor GlcNAc in situ. Importantly, no corresponding GlcNAc signals were observed outside the spots, indicating that the Carnoy’s fluid wash effectively removed endogenous GlcNAc and GlcNAc-modified lipids. A representative MS1 spectrum (Figure S4) of the observed GlcNAc-derived ions observed upon ionization of 0.5 µL of 0.1 µg/µL of the GlcNAc standard spotted on control mouse tissue illustrated the necessity for 240k resolution in this lower *m*/*z* range.

**Figure 3:**
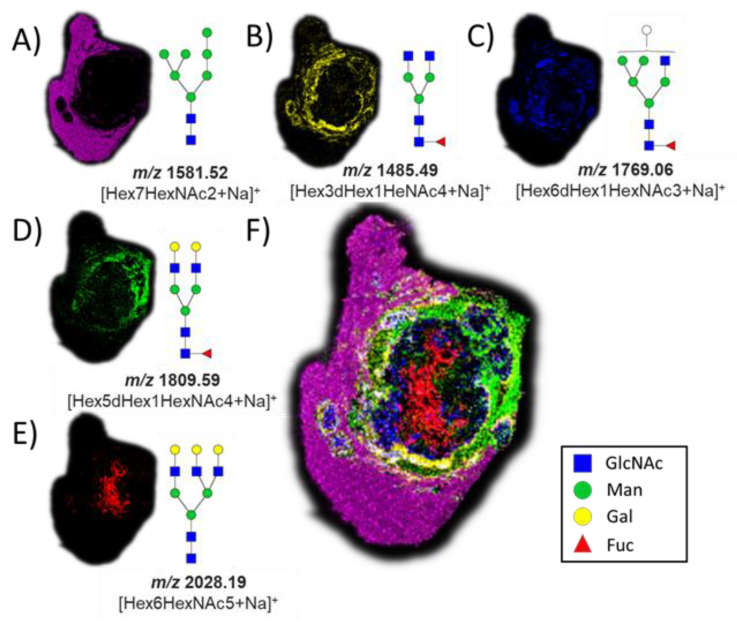
Ion maps of numerous N-glycans were detected by MALDI imaging as sodium-cationized species in different parts of the tissue after PNGase F treatment. (**A**) *m*/*z* 1581.52, Hex7HexNAc2; (**B**) *m*/*z* 1485.49, Hex3dHex1HeNAc4; (**C**) *m*/*z* 1769.06, Hex6dHex1HexNAc3; (**D**) *m*/*z* 1809.59, Hex5dHex1HexNAc4; (**E**) *m*/*z* 2028.19, Hex6HexNAc5; and (**F**) co-registered collection of glycans. The hematoxylin and eosin (H&E) stain of the same tissue section is shown in Figure 4 for reference.

The successful release of GlcNAc from glycopeptides in the tissue by the application of *Bt*OGA motivated us to pursue a two-step strategy, entailing the removal of N-linked glycans via on-tissue treatment with PNGase F followed by the release of GlcNAc by the application of *Bt*OGA. PNGase F was used to cleave all N-linked glycans from glycoproteins, thus simultaneously removing confounding terminal GlcNAc residues found on some N-glycans that might otherwise be released during the subsequent OGA treatment. We observed a subset of N-glycans in different parts of the VX2 tumor tissue after the PNGase F treatment (Figure 3) with five glycans that were identified based on accurate mass measurements, all as sodium-cationized ions: Hex7HexNAc2 (*m*/*z* 1581.52), Hex3dHex1HeNAc4 (*m*/*z* 1485.49), Hex6dHex1HexNAc3 (*m*/*z* 1769.06), Hex5dHex1HexNAc4 (*m*/*z* 1809.59), and Hex6HexNAc5 (*m*/*z* 2028.19). We also found that oligomannose N-glycans (*m*/*z* 1581.52) were abundant in the region of normal liver tissue (Figure 3A) [61]. Various fucosylated glycans were localized in the tumor region and tumor margins (Figure 3B–D), and non-fucosylated glycan was found in the necrotic regions of the VX2 tumor (Figure 3E) [62]. Additional MSI replicates on other tumor sections treated with PNGase F were consistent (Figures S5 and S6), and the entire collection of identified N-glycans is summarized in the supporting information (Table S1).

**Figure 4:**
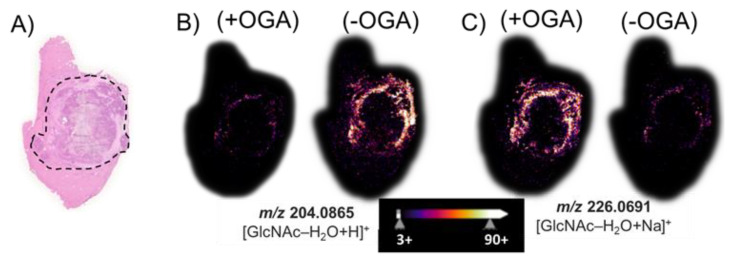
(**A**) The H&E stain of a hepatic tumor section contains an outlined tumor region composed of necrotic tumor with viable tumor region found at the interface to the healthy tissue. MALDI imaging of (**B**) the water-loss oxonium ion of GlcNAc, *m*/*z* 204.0865; and (**C**) the sodium-cationized water-loss oxonium ion, *m*/*z* 226.0691. (+) indicates the use of OGA, while (−) indicates an untreated serial section. The tissue sections were treated with PNGase F to remove all N-glycans prior to the OGA (+/−) treatment.

After we performed an MSI of tissue treated with PNGase F, we removed both the matrix and released N-glycans using a 70% ethanol washing step, and then, we treated the same VX2 tumor tissue sections with *Bt*OGA to release GlcNAc from O-GlcNAcylated proteins. The hematoxylin and eosin (H&E) stain of a hepatic tumor section revealed (Figure 4A) black outlined tumor region composed of a necrotic core with a viable tumor region found at the interface to healthy tissue. Figure 4B illustrates the non-enzymatically produced water-loss oxonium ion of GlcNAc, *m*/*z* 204.09. This GlcNAc water-loss oxonium ion (*m*/*z* 204.09) is observed in the *Bt*OGA negative control in the region surrounding the viable tumor region found at the interface to healthy tissue. This GlcNAc water-loss oxonium ion signal is produced without OGA treatment by fragmentation induced during MALDI MS analysis of samples containing O-GlcNAcylated proteins. MALDI imaging based on monitoring *m*/*z* 226.07, the sodium-cationized water-loss oxonium ion of GlcNAc is only detected in significant abundance after the *Bt*OGA treatment (Figure 4C). Additional MSI replicates on other tumor sections (Figures S7 and S8) confirmed the localization of GlcNAc to viable tumor regions. Comparisons of the abundances and distributions of these two oxonium ions (Figure 4B,C) indicated that protonated GlcNAc water-loss oxonium ions (*m*/*z* 204.09) are observed in tissue without *Bt*OGA treatment (negative controls), whereas sodium-cationized water-loss oxonium ions (*m*/*z* 226.07) are only observed after *Bt*OGA treatment and confidently demarcate the tumor margins. These results confirm that higher O-GlcNAc levels are localized to areas of cellular proliferation and malignancy as reported in HBP-focused metabolism [63]. Moreover, these data indicate that this two-step methodology faithfully allows visualizing the abundance and distribution of O-GlcNAc without interference from terminal GlcNAc of N-glycans.

To obtain information about the potential glycoproteins from which the GlcNAc signatures originated, we next performed targeted on-tissue tryptic digestion of the same serial section in areas identified as rich in GlcNAc signals (tumor regions) versus those with little or no GlcNAc signal representative of healthy tissue. The strategy entailed performing in situ proteolytic digestion by laying onto the tissue a series of polyacrylamide gel plugs (1.5 mm diameter) permeated with trypsin. The imaging results were used to direct the placement of the trypsin-soaked gel plugs onto serial sections of tissue (Figure S9A) in order to provide spatial correlations between the GlcNAc analysis and glycoproteomic analysis. In total, 14 gel plugs were placed over tissue identified as healthy tissue, and 14 gel plugs were placed over viable tumor or areas defined as tumor margins (Figure S9B). After a four-hour digestion period, we combined each of the two groups of gel plugs and then extracted the tryptic peptides and performed proteomic analyses using nanoLC-MS/MS with an HCD-triggered-UVPD method. 

Six technical replicates of each combined set of gel plugs (healthy versus tumor) were performed. The collection of results was evaluated using Proteome Discoverer (PD) for label-free quantitation and PMI Byonic for characterization of post-translational modifications (PTM). In total, 2626 proteins were identified in at least three of the six technical replicates of either healthy or tumor tissue and based on identification of at least two unique peptides (Supplementary Table S5). Based on 1,335,448 spectra for the combined six replicates of healthy and tumor tissue, we identified 315,707 peptide spectral matches corresponding to 27,945 peptides and 9881 proteins. Among the thousands of peptides identified, we found 19 were O-GlcNAcylated peptides corresponding to the same number of O-GlcNAcylated proteins observed in Proteome Discover (PD). The set of O-GlcNAcylated peptides was investigated in more detail using analysis with PMI Byonic [64]. Principal component analysis (PCA) was used to compare and dissect the variations between all proteins found in the healthy and tumor tissue datasets (Figure S10). A cluster was observed for the tumor samples that corresponded to a distinction between healthy and tumor derived tryptic peptides. Interestingly, the six healthy tissue samples showed a clear deviation from the tumor clusters; however, they did not form a cohesive cluster. A volcano plot (Figure S11) shows the distribution of proteins identified from the gel plugs based on the abundance ratio of each protein in disease versus healthy tissue for those proteins with *p* values < 0.05. The six replicates yielded 23,042 peptide spectral matches corresponding to 1605 peptides and 229 proteins significantly downregulated in tumor tissue (Table S6). Conversely, 17,537 peptide spectral matches corresponding to 1265 peptides and 217 proteins were significantly upregulated in tumor tissue (Table S7). The proteins that have significant fold decreases in the tumor tissue are mostly those associated with oxidoreductase activity, whereas proteins involved in the upregulation of metabolic processes and cell population proliferation showed significant fold increases within tumor tissue. Of the 19 O-GlcNAc-modified peptides identified by Proteome Discoverer, we matched 15 to specific glycoproteins (Table S2) using PMI Byonic. These peptides were matched based on constraints of 10 ppm mass error relative to theoretical masses of the glycopeptides and a PEP score of >300. While a common thread was not readily observed among the resulting group of O-GlcNAcylated proteins, the proteins observed are ones involved in signal transduction, cell proliferation, transport, and transcription typically regulated by phosphorylation [65]. While many of the proteins (Table S2) are reported to be implicated in cancer, calnexin, an ER chaperone protein, is significantly upregulated in several tumor phenotypes and thus used as a sero-diagnostic marker for several cancers [66]. O-GlcNAc is commonly found exclusively in the cytoplasm, nucleus, and the mitochondria of cells. However, recent work find increased O-GlcNAc may affect the regulation of vesicle formation, accelerating transport through the ER-Golgi secretory pathway [67,68].

**Figure 5:**
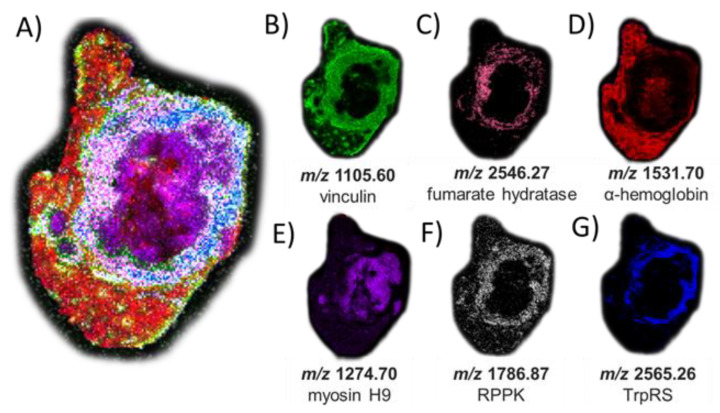
MSI of peptide signatures observed after on-tissue PNGase F and OGA treatments and trypsin digestion (and matched to peptides characterized using in-situ gel trypsin LCMS/MS analysis that correspond to known proteins). (**A**) Co-registered collection of peptide matches. (**B**) *m*/*z* 1105.60 (−0.9 ppm error) corresponding to vinculin; (**C**) *m*/*z* 2546.27 (−6.5 ppm error) corresponding to fumarate hydratase; (**D**) *m*/*z* 1531.70 (−8.9 ppm error) corresponding to hemoglobin alpha; (**E**) *m*/*z* 1274.70 (−0.1 ppm error) corresponding to myosin heavy chain 9; (**F**) *m*/*z* 1786.87 (−6.1 ppm error) corresponding to ribose-phosphate diphosphokinase; and (**G**) *m*/*z* 2565.26 (6.2 ppm error) corresponding to aminoacyl tryptophan-tRNA synthase. The H&E stain of the same tissue section is shown in Figure S8 for reference and matched peptide sequences and protein identities are reported in Table S3.

The peptides and proteins identified by LC-MS/MS were then used to guide MALDI-based imaging analysis of the same serial tissue section after on-tissue tryptic digestion. We thus mapped the locations of these peptides by MALDI MSI (Figure 5). While, as expected given the efficiency of the digestion conditions, we found no O-GlcNAc-modified peptides by MALDI imaging after on-tissue *Bt*OGA action, a collection of peptides representing abundant proteins in tumor tissue, such as α-hemoglobin and myosin heavy-chain 9, were identified (Table S3) indicating a high glycemic status at the time the tissue was collected [69]. Interestingly, one peptide (*m*/*z* 1105.60) originating from phosphoprotein vinculin was found in high abundance in the tumor margins (Figure 5B), positively correlated with the defined cell–cell adhesion in VX2 tumors and often downregulated metastatic tumorigenesis and displaying the intermodulation between phosphorylation and O-GlcNAcylation [70,71,72]. Figure 5C displays the peptide observed from Fumarate hydratase (FH) (*m*/*z* 2546.2720, THTQDAVPLTLGQEFSGYVQQVK) involved in generating energy through the Krebs cycle. O-GlcNAcylation of FH is found at phosphorylation sites under robust O-GlcNAc transferase (OGT) activity observed in the aberrant cancerous cell conditions promoting tumorgenesis [73,74]. Localization of a peptide of *m*/*z* 2565.24 in viable tumor regions was matched to DMNQVLDAYENKKPFYLYTGR from aminoacyl tryptophan-tRNA synthetase (Figure 5G). This enzyme was recently identified as a possible therapeutic target due to its immunosuppressive roles that are exploited by tumors to afford protection against immune response [75,76]. Additional MSI replicates of other tumor sections are shown in Figures S12 and S13. While protein assignments used a curated mass list of peptides matched with a 10 ppm mass error, an alternative strategy could be performed using MALDI MS/MS to identify peptides directly and unambiguously in an imaging mode and this would not require relying on LC-MS/MS data for peptide characterization.

## 3. Materials & Methods

### 3.1. Chemicals

Analytical grade acetonitrile (ACN), methanol, ethanol, and xylene were acquired from Fisher Scientific (USA). The following chemicals were also used: α-cyano-4-hydroxycinnamic acid 98% (CHCA) (Sigma-Aldrich, St. Louis, MI, USA), chloroform (Acros Organics, Geel, Belgium), ammonium bicarbonate (Sigma), acetic acid (Sigma), and trifluoroacetic acid (TFA) (Sigma-Aldrich). In situ and gel-plug digestion used Trypsin (Pierce), and PNGase F (Bulldog Bio) was used for enzymatic released of N-glycans. N-terminally 6X His-tagged Bacteroides thetaiotaomicron O-GlcNAcase (Uniprot: Q89ZI2) was produced and purified as described previously [59].

### 3.2. Sample Preparation & Analysis

Control mouse or VX2-tumors from rabbit liver tissues were mounted using a small drop of optimal cutting temperature (OCT) compound and cryosectioned using a Thermo NX50 Cryostat (Thermo Fisher Scientific, Waltham, MA, USA) at −15 °C into 12 µm-thick sections. Serial sections were thaw-mounted onto separate glass microscope slides and placed in a cabinet desiccator before tissue sections were washed with graded ethanol and Carnoy’s fluid to remove endogenous metabolites and free GlcNAc in the tissue [77]. For each sample, a serial section was subjected to hematoxylin and eosin histological staining to visualize tissue disease pathology. Optical images of the tissue sections were recorded using a Hamamatsu NanoZoomerSQ slide scanner (Japan) at 20× magnification and exported at 5× magnification.

Serial sections on separate glass slides were coated with OGA using an HTX M5 sprayer (HTX Technologies LLC, Chapel Hill, NC, USA) with parameters summarized in Table S4. OGA quality was assessed by native mass spectrometry. OGA, often found as a dimer, is highly conserved among species, and Figure S14 shows the MS1 spectrum of bacterial O-GlcNAcase *Bt*GH84. The dominant ions (25+ to 30+ charge states) correspond to a 168 kDa dimer, indicating a folded native-like form of OGA [78]. Incubation in a humidity chamber is a crucial step in each enzymatical-mediated protocol. Figure S15 shows the sealed humidity chamber constructed in-house, and the panels describe how using a heating pad kept 2–3 °C above the incubation temperature minimizes condensation from delocalizing the molecular targets of interest upon wetting the tissue section. The OGA-coated issues were incubated for two hours in this sealed humidity chamber at 30 °C. 

MSI analysis of the liver tumor sections was performed using an AP/MALDI (ng) UHR ion source (MassTech, Inc., Columbia, MD, USA) coupled with an Orbitrap Fusion Lumos Tribrid mass spectrometer (Thermo-Fisher Scientific, USA) operated in the positive ion mode. For imaging, the AP/MALDI source was operated using a constant speed raster mode at 20% laser power and a continuous raster with a 1 kHz repetition rate. Control mouse liver and rabbit VX2 tumor sections were analyzed in positive ion mode at 100 µm spatial resolution with the laser focused to generate 33 µm wide scan lines optimized to prevent over-ablation (see Figure S3). The AP-MALDI source stage was set at a velocity of 0.08 mm/second, allowing 1250 laser shots/pixel during each 1.25 s/pixel acquisition. MS spectrum imaging settings were as follows: 500 ms maximum injection time; mass range: *m*/*z* 150–600 (*m*/*z* 600–4000 for released N-glycan and peptide imaging); mass resolution: 240k at *m*/*z* 400 for GlcNAc analysis and 60k at *m*/*z* 400 for released N-glycan and peptide imaging. A 4e6 MS1 automatic gain control setting was used (1000% normalized AGC target) with a single microscan. An amount of 4.8 kV source voltage, 340 °C ion transfer tube temp, and an RF lens of 40% (120% for released N-glycan and peptide imaging) were used. 

The peptide TAB1 (sequence PVSVPYSSAQSTS) was subjected to OGT-catalyzed glycosylation to generate the O-GlcNAcylated glycopeptide (as previously described) [79] and was subsequently used for the OGA reaction optimization. Control mouse liver was used to evaluate the effectiveness of the OGA reaction on tissue (BioIVT, Westbury, NY, USA). Data analysis and visualization were performed with FreeStyle 1.8 and SCiLS Lab 2022b (Bruker, Billerica, MA, USA), and GlycoDigest (Expasy). All MSI images were root mean square normalized, meaning normalization of peak intensities in a pixel by the observed median intensity of the *m*/*z* of interest [80].

To determine a lower limit of detection for GlcNAc, a series of GlcNAc solutions were prepared with concentrations ranging from 0.005 µg/mL to 1 µg/mL. 0.5 µL of each solution was spotted on tissue sections (pre-washed with Carnoy’s solution to mitigate interference from O-GlcNAc-modified lipids or other endogenous N-acetylhexoamines) (Figure S2). The on-tissue limit of detection considers the mass quantity of the GlcNAc standard deposited on a calculated cubic mass of sectioned tissue. A MALDI resolution of 100 µm was confirmed with a raster variation of ±1.5 µm (Figure S3). Each spot in Figure S2C–E was measured as a circle with a diameter of 15 pixels (1.5 mm). The cubic volume of tissue was then calculated using the 12 µm thickness of the sectioned liver tissue. Using a tissue density of 1.05 g/cm^3^, the mass of tissue was estimated as 22.3 µg per spot. GlcNAc signal was observed down to the 5 ng/mL level (spot #6) based on detection of *m*/*z* 226.07 corresponding to the sodium-cationized water-loss oxonium ion of GlcNAc. A detection limit of 110 ng/g was determined by dividing the mass of the GlcNAc spike (i.e., 0.5 µL of 5 ng/mL solution, corresponding to 2.5 ng) by the calculated tissue mass (22.3 µg). As shown in Figure S3, at a 100 µm spatial resolution, the continuous 1 kHz raster of the AP/MALDI laser creates lines that are 33 µm wide, desorbing only a third of each constitutive pixel. As a result of this raster pattern, the limit of detection for GlcNAc oxonium ions was calculated to be a third of the spotted level or 37 ng/g. Importantly, no GlcNAc signal was observed outside the areas of the spots, indicating the Carnoy’s wash effectively removed endogenous GlcNAc and O-GlcNAc-modified lipids.

### 3.3. LC-MS/MS Analysis

For in situ tissue peptide extraction, a P1000 pipet tip was used to cut out 1.5 mm diameter plugs from Novex™ 4–20% tris-glycine gels. Plugs were placed into a microcentrifuge tube and dehydrated using three washes of 60% ACN in 100 mM ammonium bicarbonate followed by 5 min of sonication to remove any tris-glycine buffer. A final wash with 100% ACN fully dehydrated the gel plugs, which were subsequently rehydrated with 0.045 µg/µL trypsin in 100 mM ammonium bicarbonate, pH 7.8. The trypsin-laden gel plugs were carefully placed onto either the healthy or outlined viable tumor regions identified in the 12 µm thick Carnoy’s fluid washed liver tumor sections (Figure S9) and incubated at 37 °C for 4 h. After digestion, each grouping of gel plugs was collected into separate microcentrifuge tubes. Following heat inactivation for 10 min at 80 °C to quench the digestion, tryptic peptides were extracted from the polyacrylamide gel using 0.1% formic acid in 70% acetonitrile and dried under medium heat to concentrate sample. Samples were reconstituted in 0.1% formic acid, 2% acetonitrile in water before nanoLC-MS/MS analysis [81,82]. 

Six separate technical replicates of disease and healthy tissue peptide extraction digests were separated using a Dionex Ultimate 3000 nano liquid chromatography (LC) system (Thermo Scientific) plumbed for direct injection onto a 20 cm C-18 (3 μm, 300 Å pore size, 75 um ID, packed in-house) Picofrit analytical column (New Objective, Woburn, MA, USA). Mobile phases A and B were composed of HPLC-grade water and acetonitrile, respectively, each containing 0.1% formic acid. Separations were carried out using optimized gradients for various samples: injection and loading at 2% B for 5 min followed by a linear gradient from 15% to 75% B in 40 min was used for analysis of the tryptic digests from the healthy and disease tissue samples. The flow rate was maintained at 0.300 μL/min. Eluted peptides were analyzed in positive polarity mode using an Orbitrap Fusion Lumos Tribrid mass spectrometer (Thermo Fisher Scientific, San Jose, CA) equipped with a 193 nm laser for UVPD and a NanoFlex electrospray source. All mass spectra were acquired in the Orbitrap mass analyzer using resolution settings of 60 K (at *m*/*z* 200) for MS1. All MS2 spectra were acquired in the Orbitrap at 30k resolution (at *m*/*z* 200). HCD mass spectra were collected using 28 NCE, and UVPD was performed in the high-pressure linear ion trap using two 5 ns laser pulses (1.5 mJ per pulse) from the 193 nm, 500 Hz excimer laser (Coherent ExciStar).

A nano-ESI source with borosilicate emitters fabricated in-house and coated in Au/Pd was used for native MS1 analysis of OGA. The source voltage was 1.3 kV, and the source was set at a temperature of 200 °C to transfer proteins into a Thermo Scientific Q Exactive UHMR (Ultra-High Mass Range) Hybrid Quadrupole-Orbitrap mass spectrometer (Bremen, Germany) using an in-source trapping voltage of −150V to assist desolvation. MS1 spectra was based on 200 transients and a scan range of *m*/*z* 2000–12,000. Data was collected at a resolving power of 7.5K resolution at *m*/*z* 400 and a maximum ion time of 50 msec.

Data analysis and visualization were performed with FreeStyle 1.8 while Byonic (Protein Metrics, Cupertino, CA, USA) proteomic database search was used for identification and site-localization of O-linked glycopeptides with precursor and fragment mass tolerances of 10 ppm. As described previously, the main scoring metrics used to gauge the confidence of identified glycopeptides were the Delta Mod score and the PEP 2D score [79]. Label-free quantitation (LFQ) was accomplished using Proteome Discoverer (v2.4.1.15, Thermo Fisher Scientific) searched against the Uniprot database of all *Oryctolagus cuniculus* proteins with the Sequest HT search algorithm, and using a modified LFQ standard processing and consensus workflow. The processing workflow included the mass recalibration node (spectrum files RC) along with the standard spectrum selector, Minora Feature Detector, Sequest HT, and Percolator nodes. The precursor detector node was used to help minimize chimeric spectra. The precursor mass tolerance was 10 ppm, and the fragment mass tolerance was set to 0.02 Da, with two missed trypsin cleavages and variable peptide modifications allowed, including oxidized methionine and N-terminal protein modifications of Acetyl and/or Met loss. False discovery rate (FDR) tolerances in the Percolator node were set to 0.01 for high confidence and 0.05 for medium confidence. Protein abundance calculations across the entire data set were based on the summed abundance of extracted ion profiles from the collective MS1 profiles of peptides unique to the identified protein with ratios calculated using pairwise comparisons. The maximum allowed fold change was 100. Reported significant proteins were those with a *p*-value < 0.05 and greater 1-fold change. 

## 4. Conclusions

We developed a two-step procedure using an O-GlcNAc hydrolase homologue *Bt*GH84 in serial enzymatic treatment of tissue sections to release GlcNAc for MSI after initial treatment with PNGase F for N-glycan imaging. The general procedure utilized to release N-glycans for MSI has proven to be a robust method applied across animal models exhibiting diverse pathology. The in situ OGA method described here leverages and expands on this established method, allowing MSI of O-GlcNAc across nearly any type of tissue and its histopathology, whether formalin-fixed paraffin-embedded or fresh-frozen. This approach enables the mapping of O-GlcNAc by utilizing high-resolution MS for the determination of GlcNAc oxonium ion species in an *m*/*z* range congested with numerous other small molecules. O-GlcNAc was localized to both viable tumor and tumor margin regions validated by histopathology of the H&E-stained liver tumor section. These imaging results are consistent with higher O-GlcNAc levels being correlated to areas of cellular proliferation and malignancy in a manner that is evident in the malignant VX2 tumor model examined here. We used targeted on-tissue tryptic digestion using gel plugs laid onto tissue and subsequent LC-MS/MS analysis to identify glycoproteins associated with GlcNAc marker ions, thus allowing the correlation of the locations of the O-GlcNAc-derived signals with specific glycoproteins. For this aspect, MALDI imaging was used for peptide localization based on high mass accuracy analysis to allow inference of the spatial distribution of the glycoproteins. 

## Data Availability

Database outputs for LC-MS/MS data sets are included in Supplemental Material. The data that support the findings of this study are openly available in [METASPACE at https://metaspace2020.eu/project/OGlcNAc_EE].

## Supplementary Materials

The following supporting information can be downloaded at: https://www.mdpi.com/article/10.3390/cancers15041224/s1, Figure S1: Nano-ESI MS1 of 10 µM TAB1-O-GlcNAc glycopeptide standard (not OGA reacted); Figure S2: (A) Summary of the dilutions of GlcNAc applied as spots on the Carnoy’s fluid washed tissue sections. The concentrations ranged from 1.0 µg/mL to 5.0 ng/mL, and 0.5 µL was applied per 1.5 mm diameter spot. (B) The H&E stain of control mouse liver. (C) MSI of *m*/*z* 244.0803, corresponding to the sodium-cationized GlcNAc ion. (D) MSI of *m*/*z* 204.0865, corresponding to the water-loss GlcNAc oxonium ion. (E) MSI of the sodium-cationized GlcNAc water-loss oxonium ion, *m*/*z* 226.0684. The six spots are labelled according to panel (A); Figure S3: Raster pattern of tissue used in limit of detection experiment (Figure S2); Figure S4: (A) MS1 spectrum spanning *m*/*z* 200-250 to monitor endogenous ions and the various GlcNAc ions observed upon ionization of 0.5 µL of 0.1 µg/µL of GlcNAc standard spotted on control mouse tissue. (B) Expanded region showing the isotope distribution of the protonated water-loss oxonium ion of GlcNAc with a −1.2 ppm mass error relative to the theoretical monoisotopic *m*/*z* 204.0864. (C) Expanded region showing the isotope distribution corresponding to the sodium-cationized water-loss GlcNAc oxonium ion observed with a −0.9 ppm mass error relative to the monoisotopic *m*/*z* 226.0684; Figure S5: Ion maps of numerous N-glycans detected by MALDI imaging as sodium-cationized species in different parts of the tissue after PNGase F treatment; Figure S6: Ion maps of numerous N-glycans detected by MALDI imaging as sodium-cationized species in different parts of the tissue after PNGase F treatment; Figure S7: (A) The H&E stain of a hepatic tumor section contains an outlined tumor region composed of necrotic tumor with viable tumor region found at the interface to the healthy tissue. MALDI ion maps of (B) GlcNAc water-loss oxonium ion, *m*/*z* 204.0865 and (C) sodium-cationized water-loss oxonium ion, *m*/*z* 226.0691. (+OGA) corresponds to the use of OGA, while (-OGA) indicates an untreated section. The tissue was treated with PNGase F to remove all N-glycans prior to the OGA (+/−) treatment; Figure S8: (A) The H&E stain of a hepatic tumor section contains an outlined tumor region composed of necrotic tumor with viable tumor region found at the interface to the healthy tissue. MALDI ion maps of (B) GlcNAc water-loss oxonium ion, *m*/*z* 204.0865, and (C) sodium-cationized water-loss oxonium ion, *m*/*z* 226.0691. (+OGA) corresponds to the use of OGA, while (-OGA) indicates an untreated section. The tissue was treated with PNGaseF to remove all N-glycans prior to the OGA (+/−) treatment; Figure S9: (A) Serial sections of tissue used for in situ trypsin-permeated gel plug extraction. (B) Placement of the 1.5 mm plugs was directed by MSI results and tissue morphology identifying defined healthy vs. tumor regions on the VX2 tumor tissue; Figure S10: LC-MS/MS results from in situ trypsin-permeated gel plug extract were subjected to principal component analysis (PCA); Figure S11: Distribution of proteins identified from selectively placed gel plugs on VX2 tumor tissue; Figure S12: MSI of peptides observed after trypsin digestion (and matched to peptides characterized using in-situ gel trypsin LCMS/MS analysis); Figure S13: MSI of peptides observed after on-tissue trypsin digestion (and matched to peptides characterized using in-situ gel trypsin LC-MS/MS analysis); Figure S14: Native MS1 spectrum obtained for a solution containing 5 µM O-GlcNAcase BT4395 from Bacteroides thetaiotaomicron VPI-5482 in 100 mM ammonium acetate; Figure S15: (A) A 100 × 15 mm petri dish lined with Wypallx60 and two 11 × 21 cm Kimwipes. The paper towels were saturated with 10 mL of deionized H2O to maximize the humidity in the chamber once sealed. (B) Temperature controlled heating pad resting on petri dish lid. (C) Tissue section after an overnight incubation at 37 °C in the humidity chamber without a heating pad permitted condensation that saturated the tissue and caused a speckled delocalized effect. (D) Results after an overnight incubation using a heated lid; Table S1: Summary of identified N-glycans detected by MALDI MSI of PNGase F-treated tissue; Table S2: Summary of identified O-GlcNAc-modified peptides with Byonic scores > 300; Table S3: Summary of peptide identifications based on intact mass within 10 ppm and PEP scores > 300 from LC-MS/MS data set matched to MSI data; Table S4: Summary of HTX M5 sprayer parameters used for each enzymatic reaction. The track pattern can be described as crisscross (CC) or horizonal-horizontal (HH) spray patterns; Table S5: Total; Table S6: Downregulated; Table S7: Upregulated.
