# Supplementary material for: In Situ Imaging of O-Linked β-N-Acetylglucosamine Using On-Tissue Hydrolysis and MALDI Mass Spectrometry
Source: Cancers (Basel). 2023 Feb 15;15(4):1224. doi: 10.3390/cancers15041224 (PMC9954453; doi:10.3390/cancers15041224)
Supplement: Supplementary file 1 [file cancers-15-01224-s001.zip › cancers-2213912-Supplementary.pdf]

Supporting Information:

In Situ Imaging of O-linked β-N-acetylglucosamine Using On-Tissue Hydrolysis and MALDI Mass Spectrometry

Edwin E. Escobar[1], Erin H. Seeley[1], Jesús E. Serrano-Negrón[2], David J. Vocadlo[2] and Jennifer S. Brodbelt[1*]
[1]*Department of Chemistry, The University of Texas at Austin, Austin, Texas 78712, United States*
[2]*Department of Chemistry, Simon Fraser University, Burnaby, British Columbia V5A 1S6, Canada*
[*]Correspondence: J. Brodbelt jbrodbelt@cm.utexas.edu

Table of Contents

Supplementary Figures..1
Supplementary Tables..16

Supplementary Figures

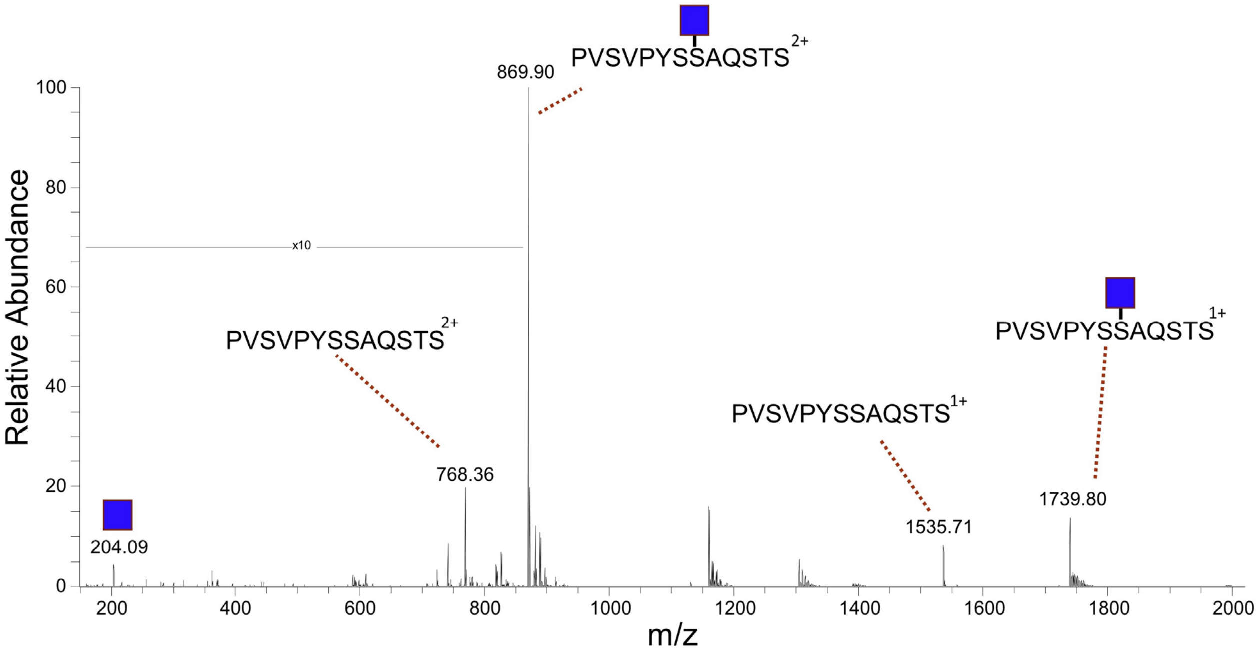

Figure S1: Nano-ESI MS1 of 10 µM TAB1-O-GlcNAc glycopeptide standard (not OGA reacted). The unmodified peptide and free GlcNAc are observed in low abundance, likely due to the intrinsic lability of the beta-O-glycosidic linkage.

A.

Spot #	Conc. (µg/mL)
1	1
2	0.5
3	0.1
4	0.05
5	0.01
6	0.005

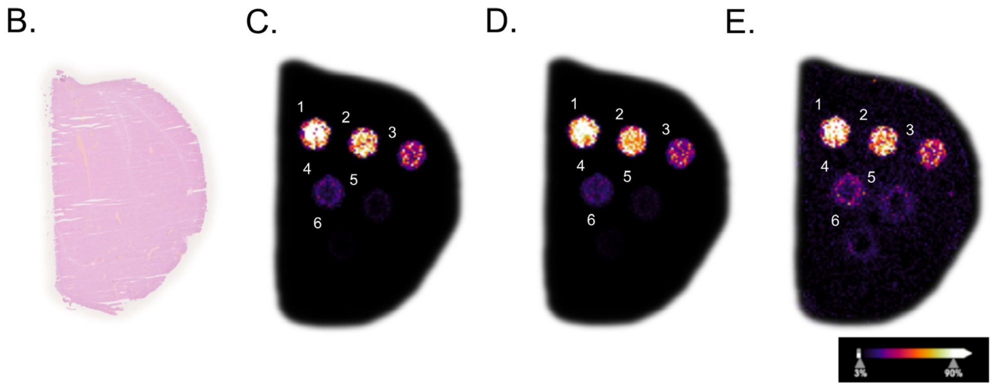

Figure S2. A) Summary of the dilutions of GlcNAc applied as spots on the Carnoy's fluid washed tissue sections. The concentrations ranged from 1.0 µg/mL to 5.0 ng/mL, and 0.5 µL was applied per 1.5 mm diameter spot. B) The H&E stain of control mouse liver. C) MSI of m/z 244.0803, corresponding to the sodium-cationized GlcNAc ion. D) MSI of m/z 204.0865, corresponding to the water-loss GlcNAc oxonium ion. E) MSI of the sodium-cationized GlcNAc water-loss oxonium ion, m/z 226.0684. The six spots are labelled according to panel A.

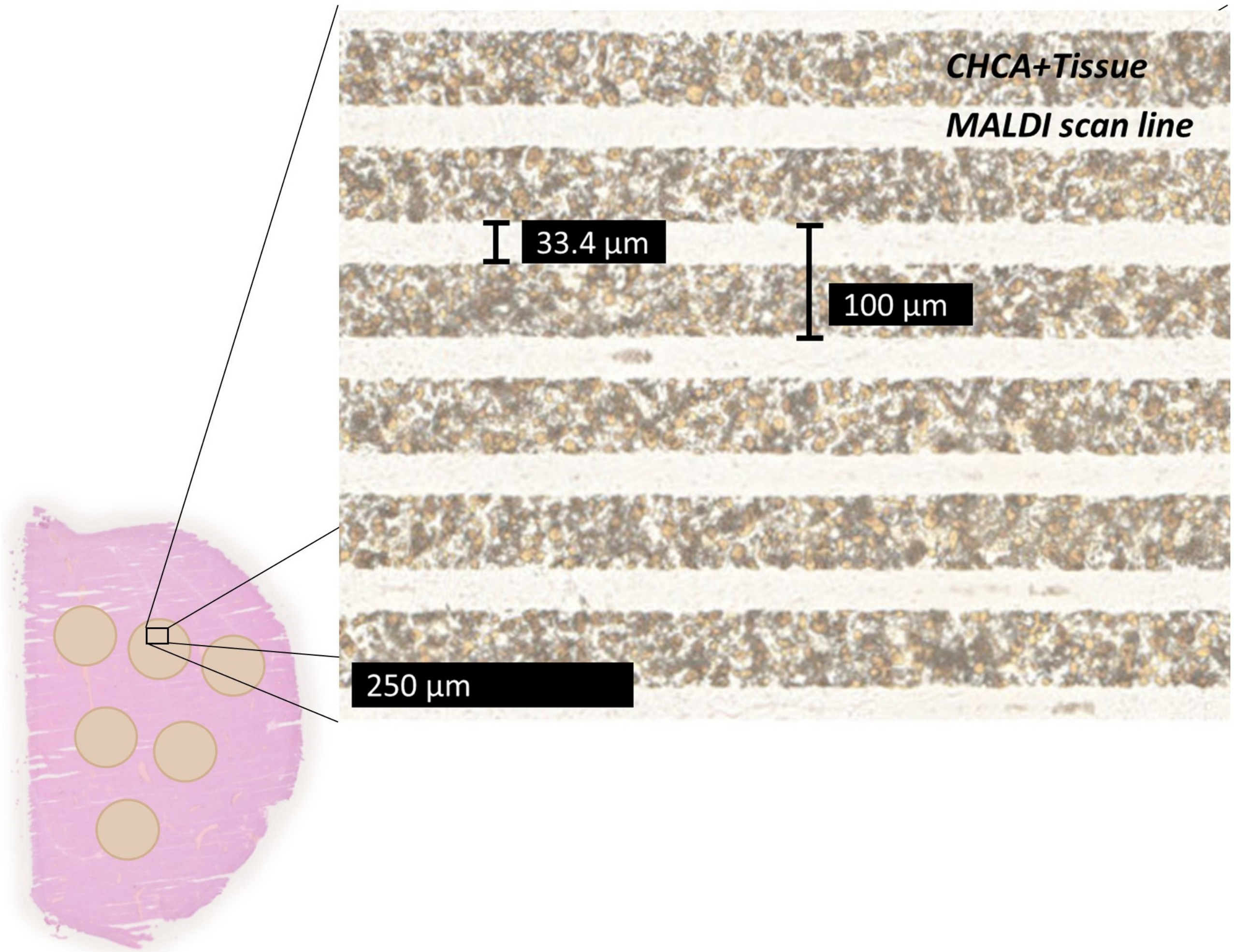

Figure S3: Raster pattern of tissue used in limit of detection experiment (**Figure S2**). At a 100 µm spatial resolution, the continuous raster pattern with a 1 kHz laser repetition rate generates 33 µm scan lines, roughly a third of each constitutive pixel. As a result of the raster pattern, the limit of detection for O-GlcNAc oxonium ions is calculated to be a third of the spotted level. Each spot (shown as tan circles positioned on the tissue on the left side of the figure) has a diameter of 15 pixels or approximately 1.5 mm.

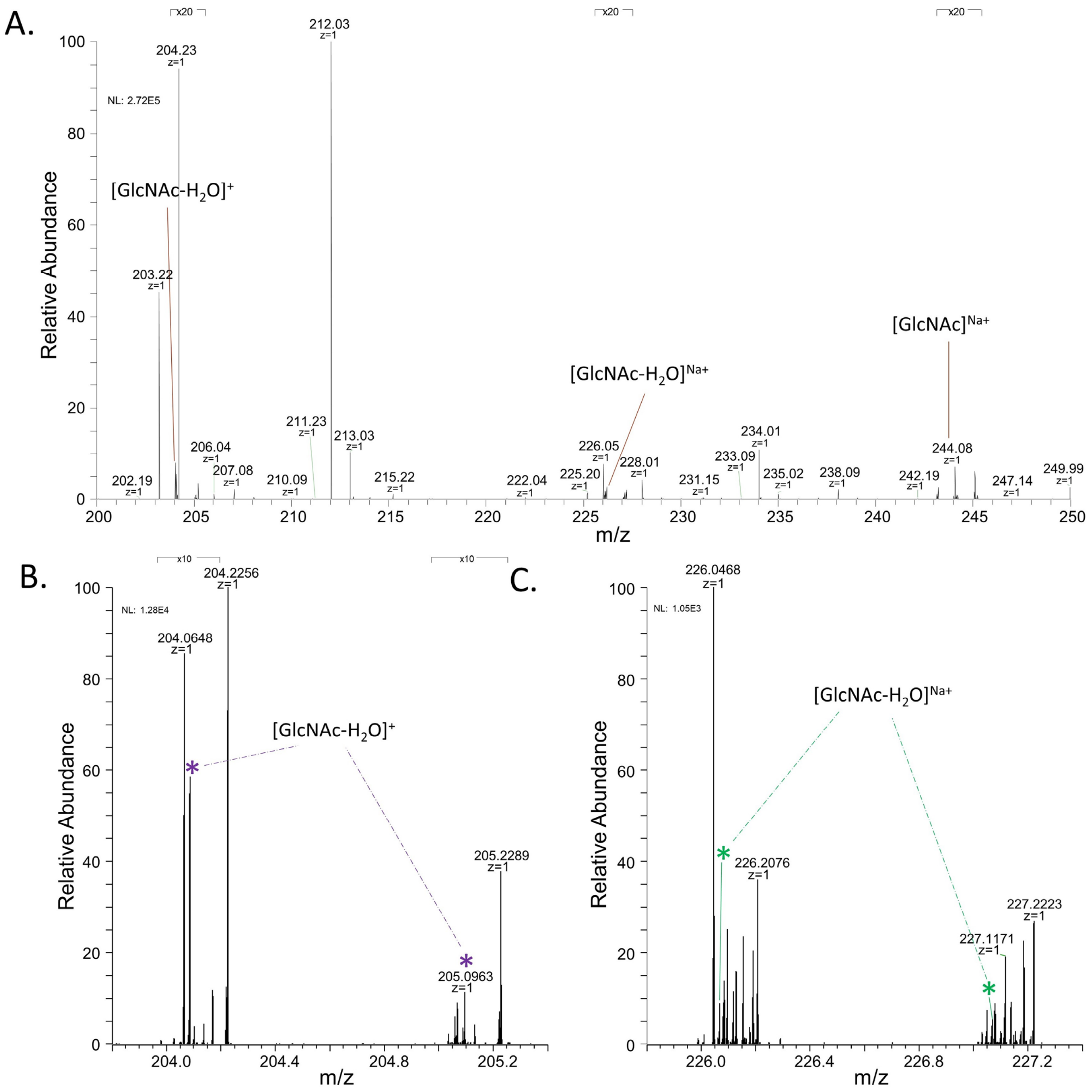

Figure S4: A) MS1 spectrum spanning *m/z* 200-250 to monitor endogenous ions and the various GlcNAc ions observed upon ionization of 0.5 µL of 0.1 µg/µL of GlcNAc standard spotted on control mouse tissue. B) Expanded region showing the isotope distribution of the protonated water-loss oxonium ion of GlcNAc with a -1.2 ppm mass error relative to the theoretical monoisotopic *m/z* 204.0864. C) Expanded region showing the isotope distribution corresponding to the sodium-cationized water-loss GlcNAc oxonium ion observed with a -0.9 ppm mass error relative to the monoisotopic *m/z* 226.0684.

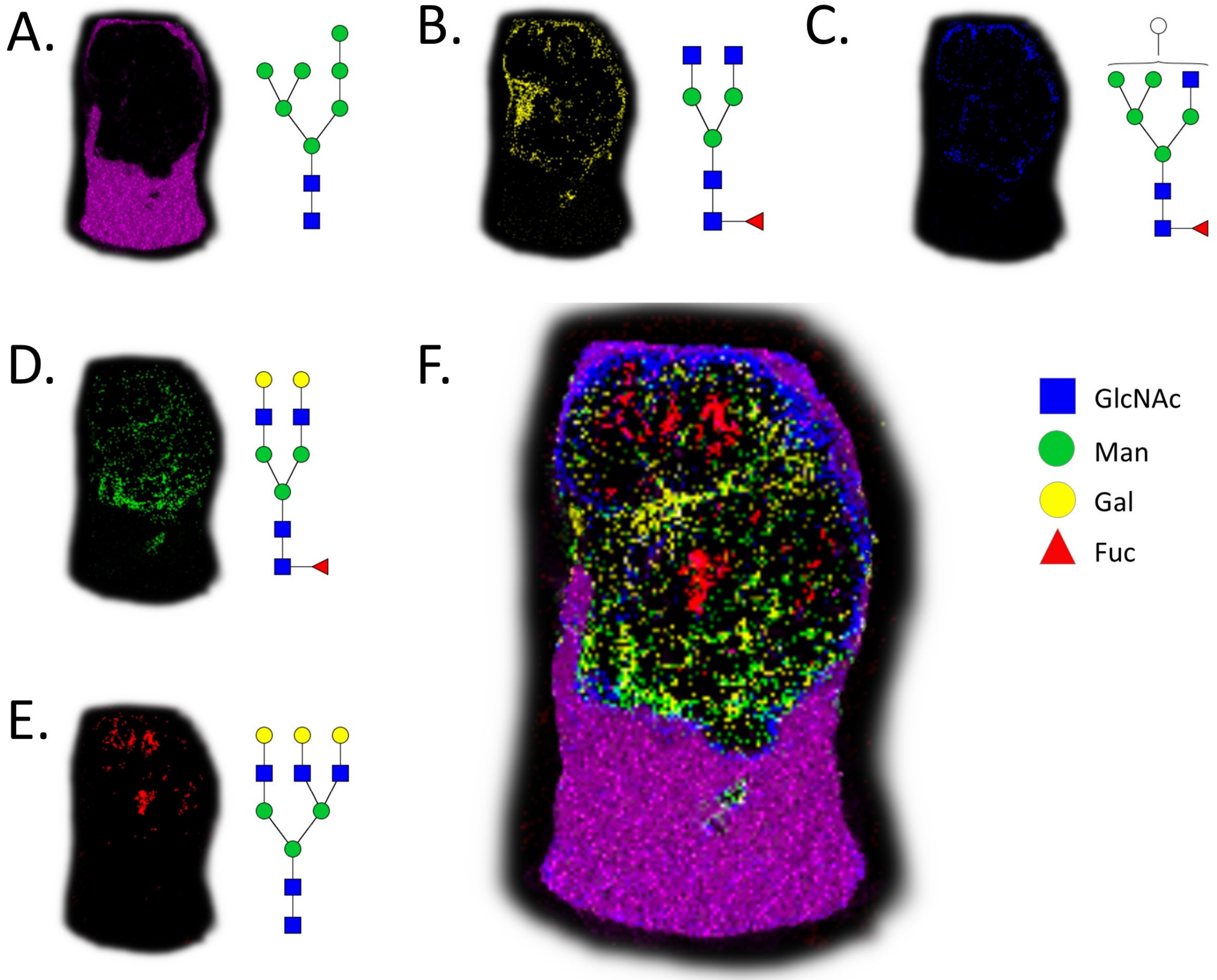

Figure S5. Ion maps of numerous N-glycans detected by MALDI imaging as sodium-cationized species in different parts of the tissue after PNGase F treatment. A) *m/z* 1581.52, Hex7HexNAc2; B) *m/z* 1485.49, Hex3dHex1HeNAc4; C) *m/z* 1769.06, Hex6dHex1HexNAc3; D) *m/z* 1809.59, Hex5dHex1HexNAc4; E) *m/z* 2028.19, Hex6HexNAc5; and F) co-registered collection of glycans. The H&E stain of the same tissue section is shown in **Figure S7** for reference.

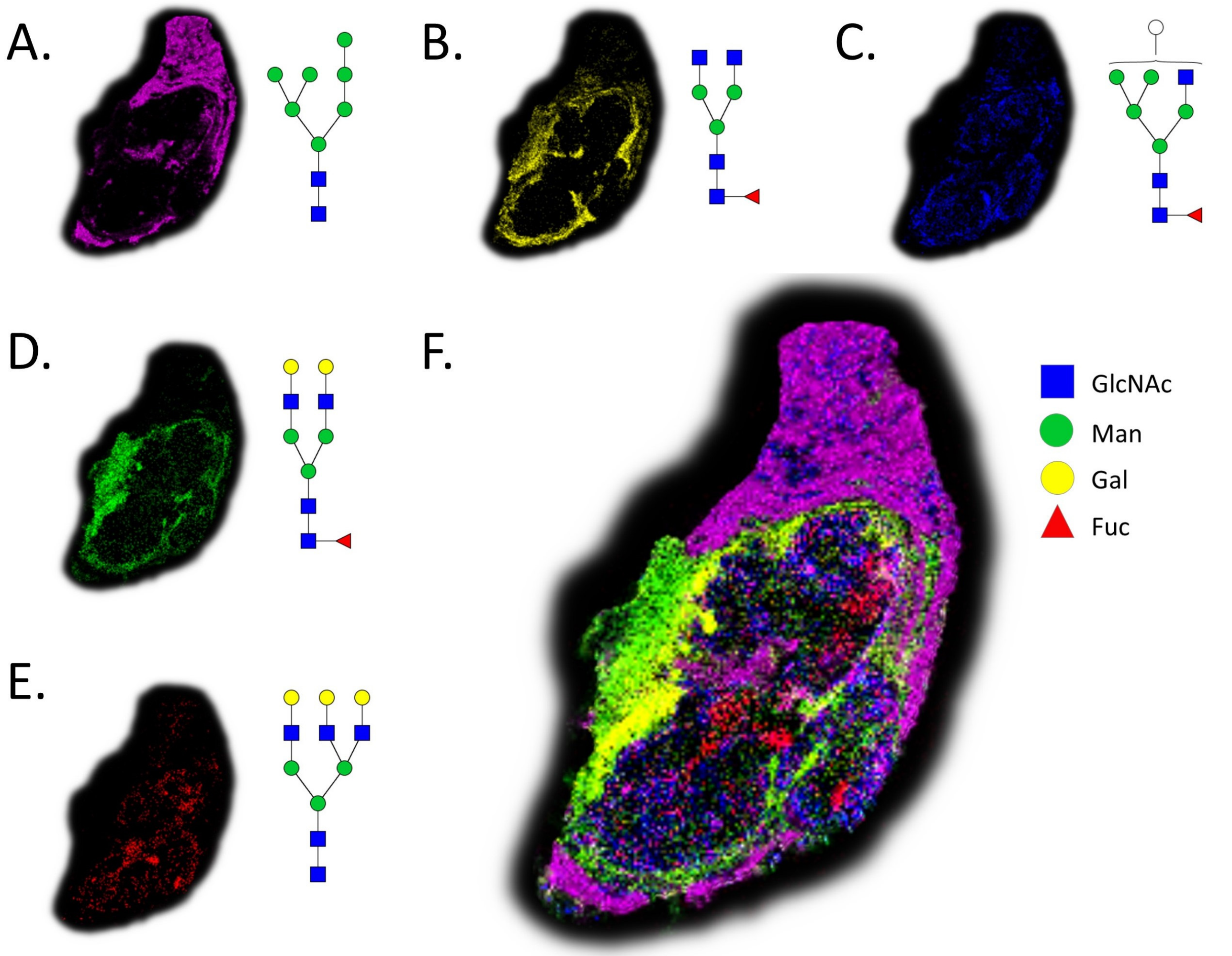

Figure S6. Ion maps of numerous N-glycans detected by MALDI imaging as sodium-cationized species in different parts of the tissue after PNGase F treatment. A) *m/z* 1581.52, Hex7HexNAc2; B) *m/z* 1485.49, Hex3dHex1HeNAc4; C) *m/z* 1769.06, Hex6dHex1HexNAc3; D) *m/z* 1809.59, Hex5dHex1HexNAc4; E) *m/z* 2028.19, Hex6HexNAc5; and F) co-registered collection of glycans. The H&E stain of the same tissue section is shown in **Figure S8** for reference.

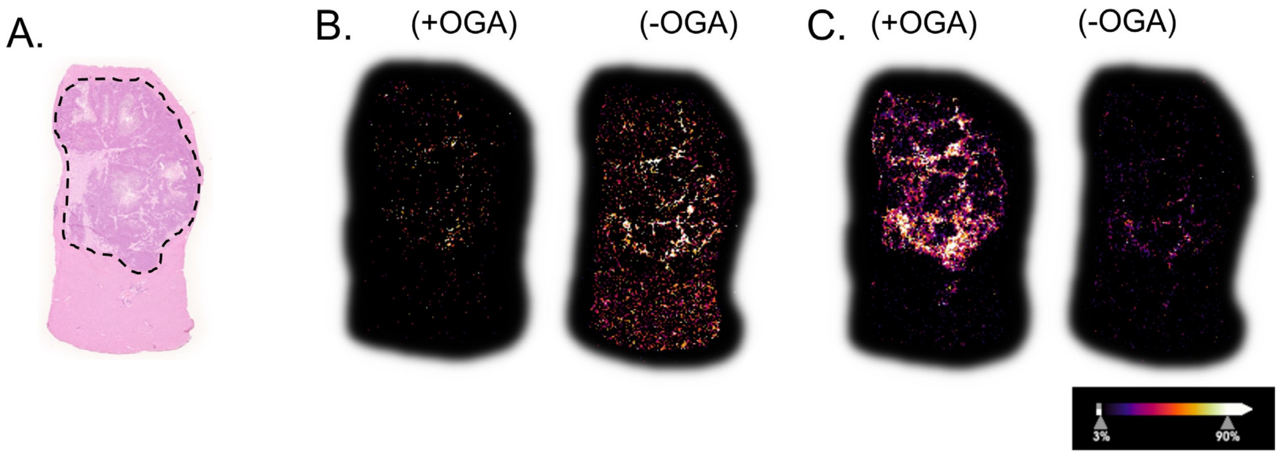

Figure S7. A) The H&E stain of a hepatic tumor section contains an outlined tumor region composed of necrotic tumor with viable tumor region found at the interface to the healthy tissue. MALDI ion maps of B) GlcNAc water-loss oxonium ion, *m/z* 204.0865 and C) sodium-cationized water-loss oxonium ion, *m/z* 226.0691. (+OGA) corresponds to the use of OGA, while (-OGA) indicates an untreated section. The tissue was treated with PNGase F to remove all N-glycans prior to the OGA (+/-) treatment.

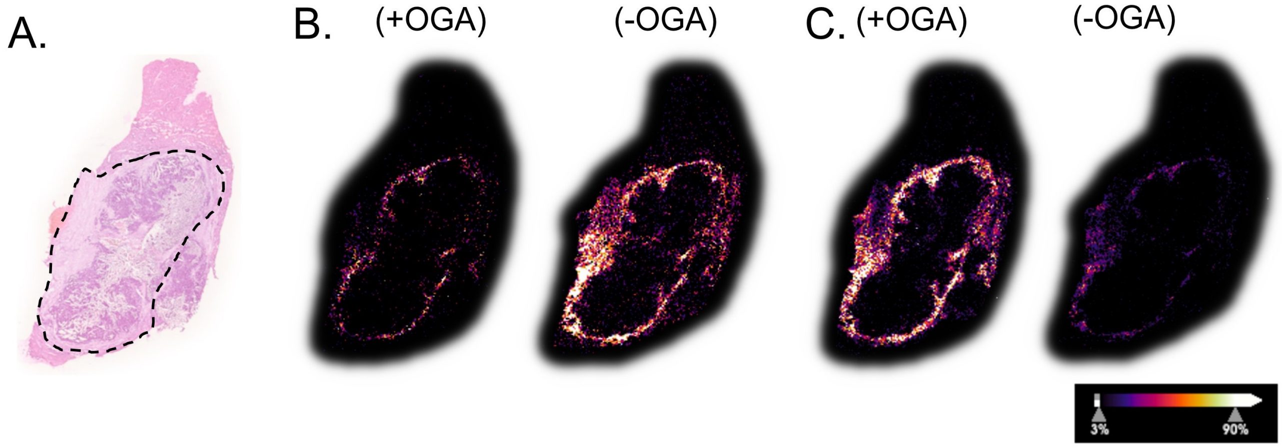

Figure S8. A) The H&E stain of a hepatic tumor section contains an outlined tumor region composed of necrotic tumor with viable tumor region found at the interface to the healthy tissue. MALDI ion maps of B) GlcNAc water-loss oxonium ion, m/z 204.0865, and C) sodium-cationized water-loss oxonium ion, m/z 226.0691. (+OGA) corresponds to the use of OGA, while (-OGA) indicates an untreated section. The tissue was treated with PNGaseF to remove all N-glycans prior to the OGA (+/-) treatment.

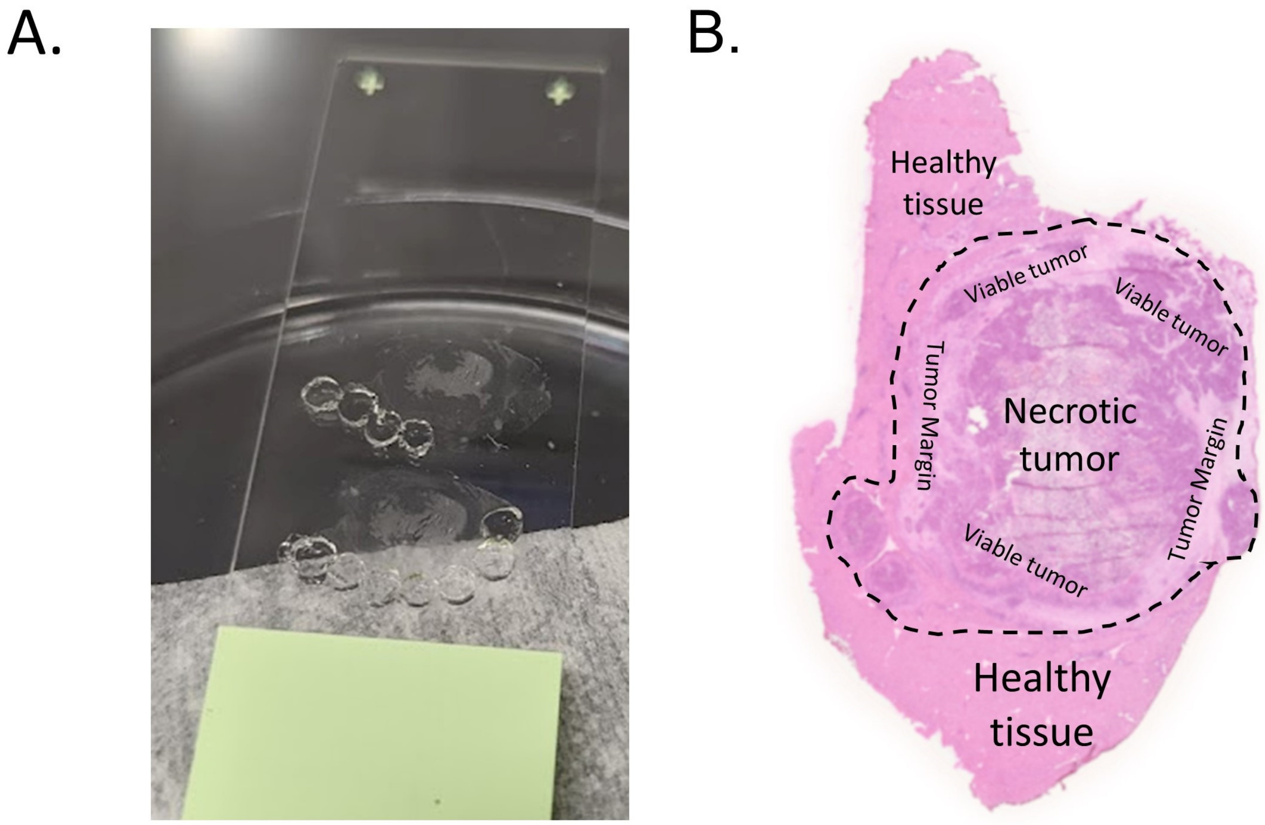

Figure S9. A) Serial sections of tissue used for in situ trypsin-permeated gel plug extraction. B) Placement of the 1.5 mm plugs was directed by MSI results and tissue morphology identifying defined healthy vs. tumor regions on the VX2 tumor tissue.

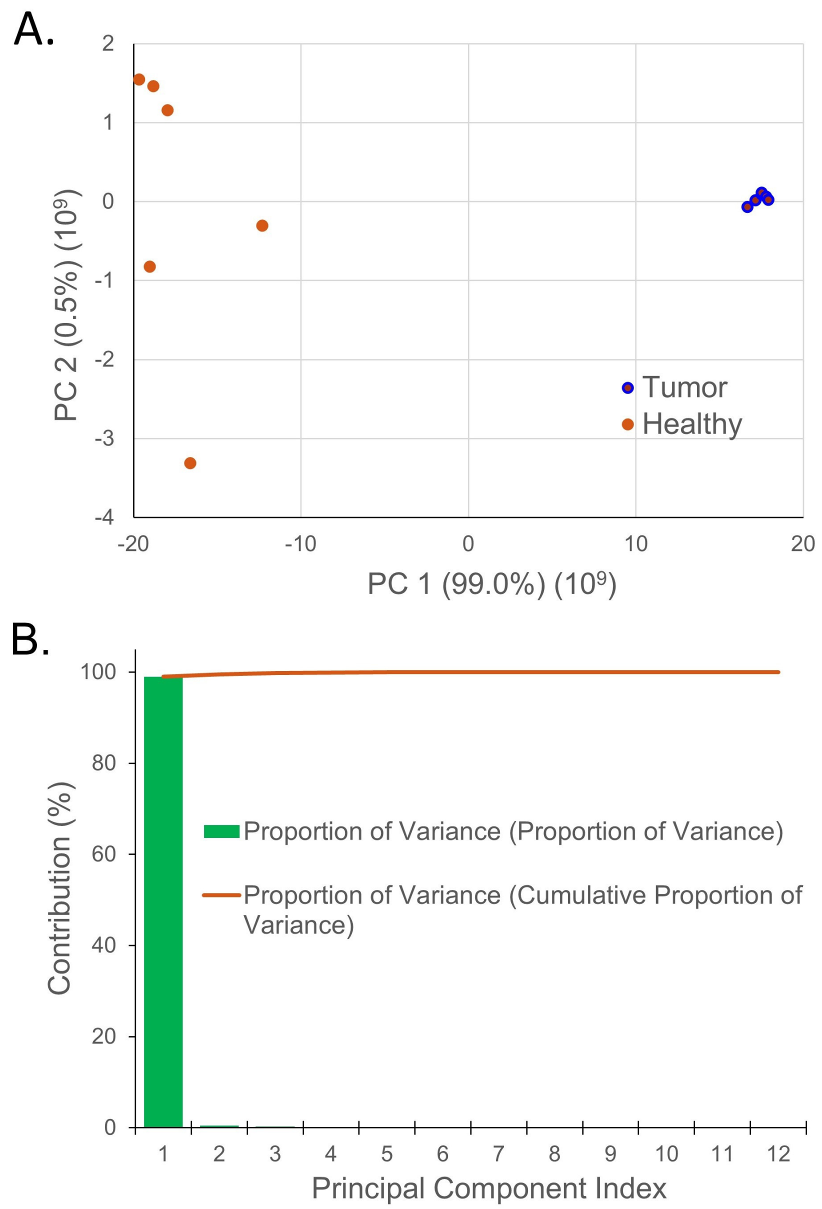

Figure S10. LC-MS/MS results from in situ trypsin-permeated gel plug extract were subjected to principal component analysis (PCA). A) PCA scores plot comparing and showing a clear distinction between the two most significant contributors to variance, principal component 1 (PC1) and principal component 2 (PC2). B) The Scree plot the assignment of nearly all contributions of variance to PC1 (99%), likely the difference between sample classes (tumor vs. healthy). PC1 and PC2 explain 99.5% of the variance observed.

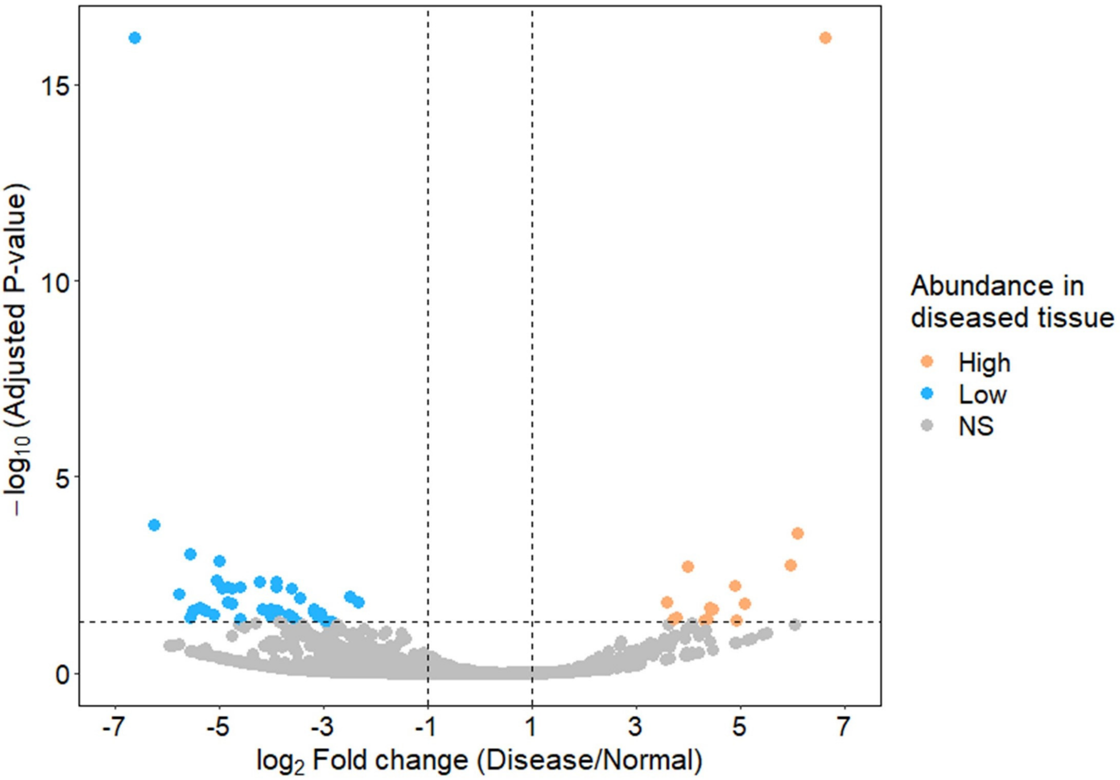

Figure S11. Distribution of proteins identified from selectively placed gel plugs on VX2 tumor tissue. The volcano plots describe each protein observed as the ratio of protein abundance in disease versus normal tissue for various positions of the gel plugs on tissue. Proteins with $p < 0.05$ are found above the horizontal dashed line. Proteins with significant fold-change are found in the regions to the left and right of the vertical dashed lines. Proteins that are higher in abundance in diseased tissue are indicated as orange circles, and those lower in abundance in diseased tissue are indicated as blue circles for each comparison. Those proteins for which the abundance was not significant are indicated as gray circles ("NS" in the legend). A list of all proteins identified is included in **Supplementary Table 5**, with low and high abundance proteins filtered and listed in **Supplementary Tables 6-7**, respectively.

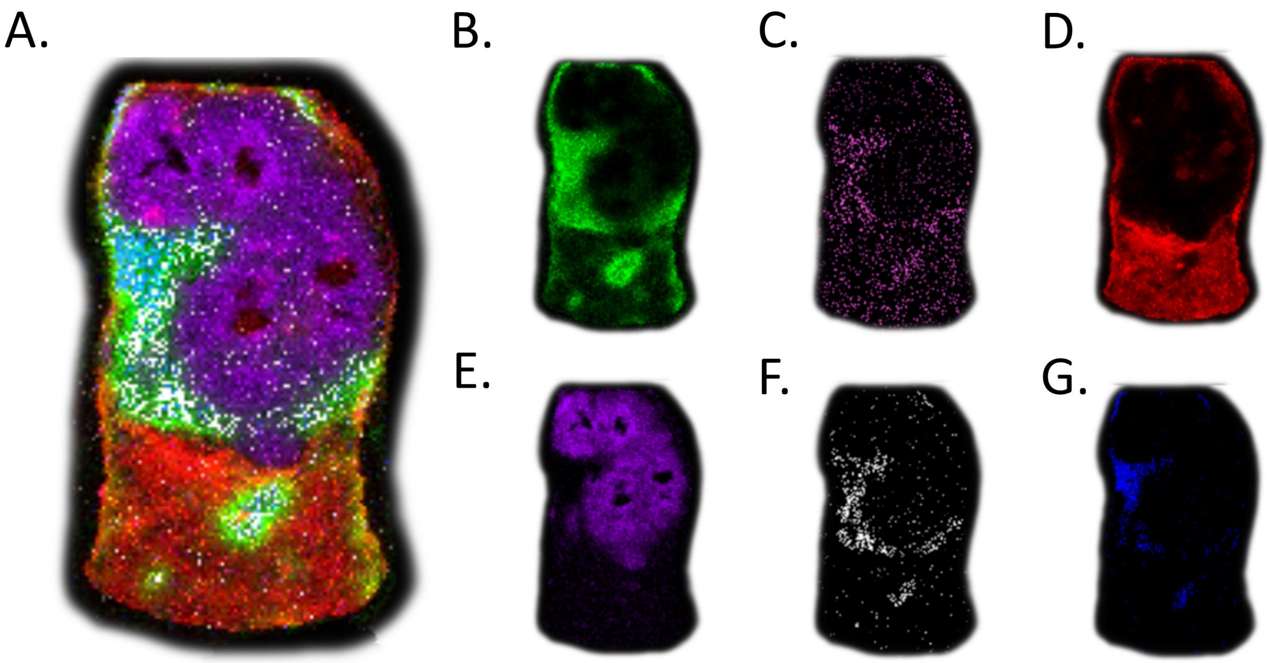

Figure S12. MSI of peptides observed after trypsin digestion (and matched to peptides characterized using in-situ gel trypsin LCMS/MS analysis). A) Co-registered collection of peptide matches. B) *m/z* 1105.60, C) *m/z* 2546.27, D) *m/z* 1274.70, E*)* *m/z* 1531.70, F) *m/z* 1786.87, and G) *m/z* 2565.26. The H&E stain of the same tissue section shown in **Figure S7** for reference and matched peptide sequences are reported in **Table S3** for reference. The tissue was treated with PNGase F and OGA prior to trypsin digestion.

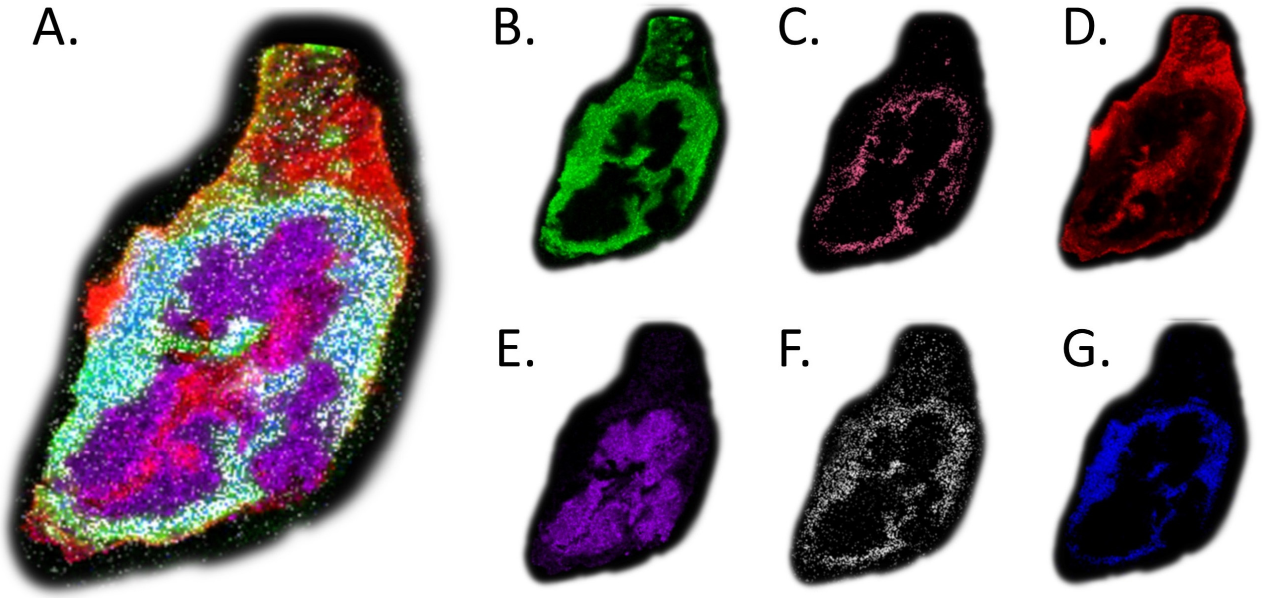

Figure S13. MSI of peptides observed after on-tissue trypsin digestion (and matched to peptides characterized using in-situ gel trypsin LC-MS/MS analysis). A) Co-registered collection of peptide matches. B) m/z 1105.60; C) *m/z* 2546.27; D) *m/z* 1274.70; E) *m/z* 1531.70; F) *m/z* 1786.87; and G) *m/z* 2565.26. The H&E stain of the same tissue section shown in **Figure S8** for reference and matched peptide sequences are reported in **Table S3** for reference. The tissue was treated with PNGase F and OGA prior to trypsin digestion.

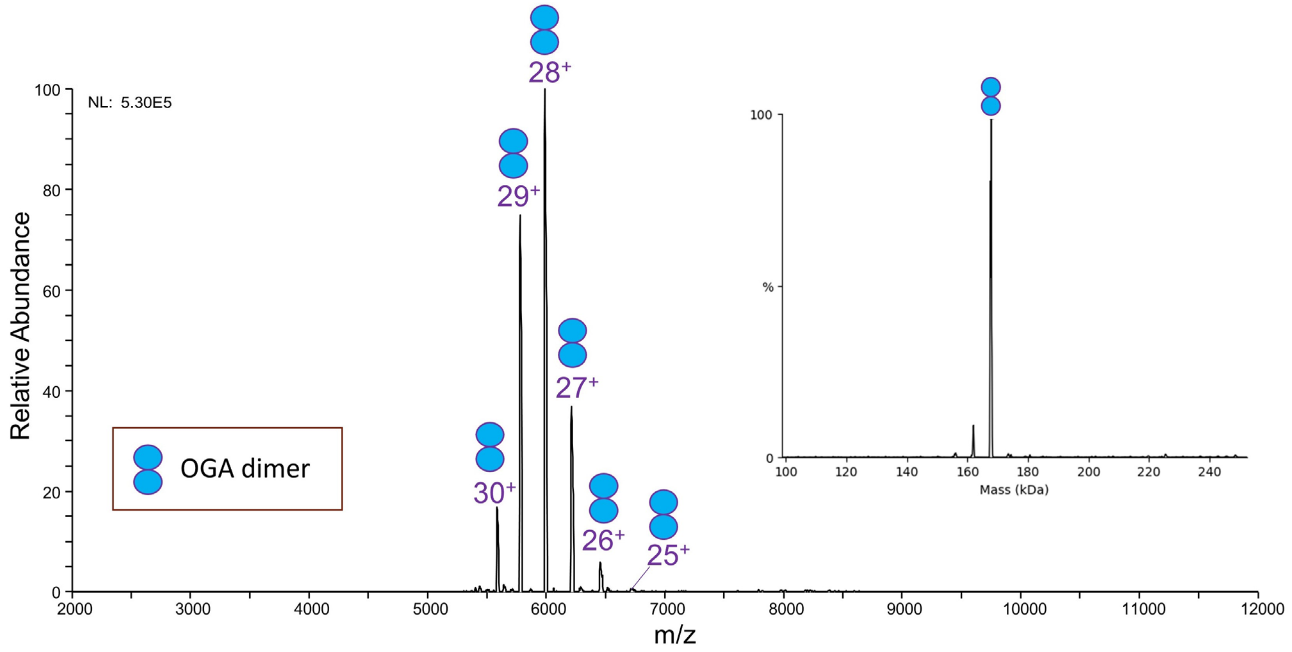

Figure S14. Native MS1 spectrum obtained for a solution containing 5 µM O-GlcNAcase BT4395 from Bacteroides thetaiotaomicron VPI-5482 in 100 mM ammonium acetate. The inset shows the deconvoluted, deisotoped mass spectrum revealing the mass of 167,900 Da (for the dimer).

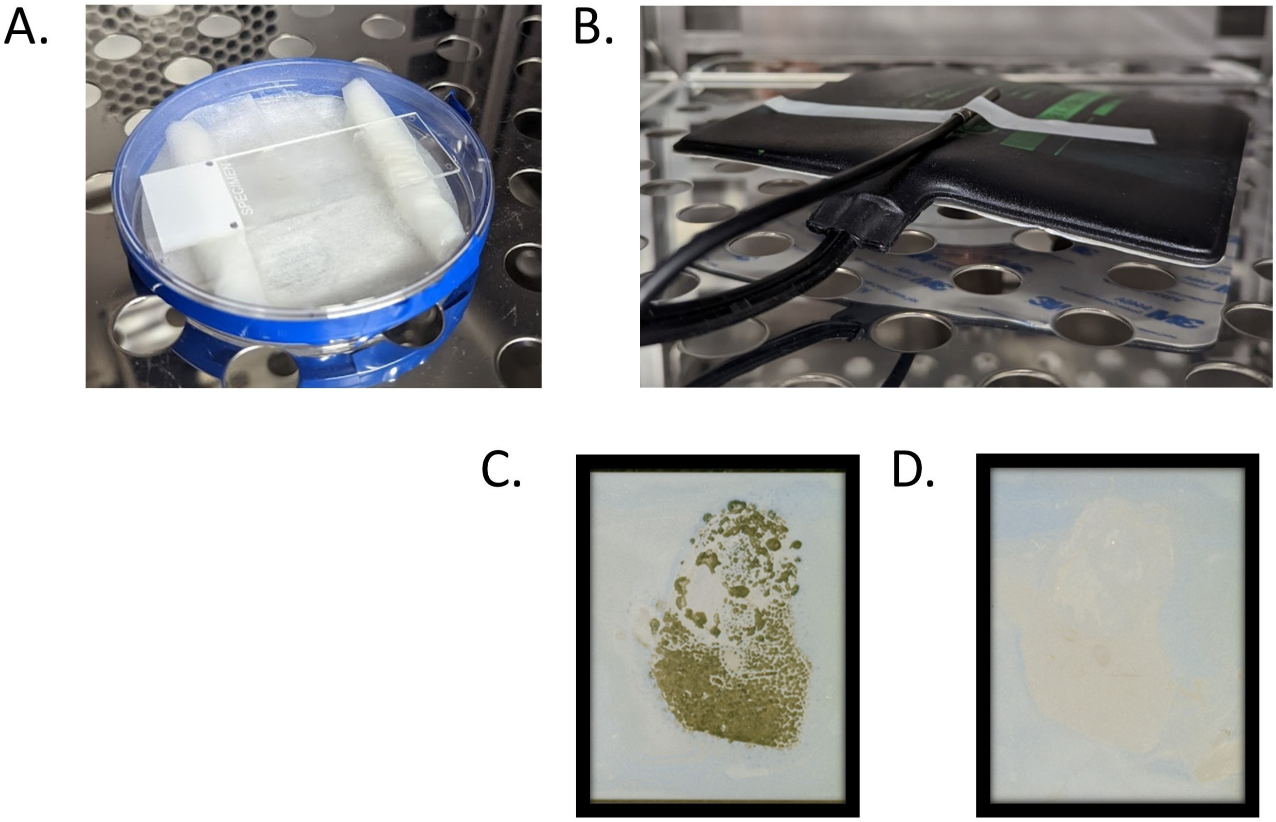

Figure S15. A) A 100 x 15 mm petri dish lined with Wypallx60 and two 11 x 21 cm Kimwipes. The paper towels were saturated with 10 mL of deionized H_2O to maximize the humidity in the chamber once sealed. B) Temperature controlled heating pad resting on petri dish lid. C) Tissue section after an overnight incubation at 37°C in the humidity chamber without a heating pad permitted condensation that saturated the tissue and caused a speckled delocalized effect. D) Results after an overnight incubation using a heated lid.

Supplementary Tables

Theoretical *m/z*	Observed *m/z*	ppm error	Glycan Composition
1079.3749	1079.3756	0.6	Hex3dHex1HexNAc2 + 1Na
1095.3698	1095.3716	1.6	Hex4HexNAc2 + 1Na
1136.3998	1136.3964	-3.0	Hex3HexNAc3 + 1Na
1282.4543	1282.4429	-8.9	Hex3dHex1HexNAc3 + 1Na
1298.4492	1298.4464	-2.2	Hex4HexNAc3 + 1Na
1444.5071	1444.4973	-6.8	Hex4dHex1HexNAc3 + 1Na
1485.5336	1485.5373	2.5	Hex3dHex1HexNAc4 + 1Na
1581.5283	1581.5147	-8.6	Hex7HexNAc2 + 1Na
1589.5446	1589.5329	-7.4	Hex4HexNAc3 + 1Na
1606.5599	1606.5593	-0.4	Hex5dHex1HexNAc3 + 1 Na
1647.5865	1647.5748	-7.1	Hex4dHex1HexNAc4 + 1Na
1663.5814	1663.5769	-2.7	Hex5HexNAc4 + 1Na
1688.613	1688.6163	2.0	Hex3dHex1HexNAc5 + 1Na
1743.5811	1743.5943	7.6	Hex8HexNAc2 + 1Na
1809.6393	1809.6463	3.9	Hex5dHex1HexNAc4 + 1Na
1905.6339	1905.6499	8.4	Hex9HexNAc2 + 1Na
1954.6768	1954.6705	-3.2	Hex5HexNAc4NeuAc1 + 1Na
1976.6588	1976.6508	-4.0	Hex5HexNAc4NeuAc1 + 2Na
2067.6866	2067.6800	-3.2	Hex10HexNAc2 + 1Na
2100.7347	2100.7325	-1.0	Hex5dHex1HexNAc4NeuAc1 + 1Na
2122.7167	2122.7081	-4.1	Hex5dHex1HexNAc4NeuAc1 + 2Na
2245.7722	2245.7584	-6.1	Hex5HexNAc4NeuAc2 + 1Na
2289.7361	2289.7224	-6.0	Hex5HexNAc4NeuAc2 + 3Na
2393.8458	2393.8440	-0.8	Hex7HexNAc6 + 1Na
2414.8325	2414.8484	6.6	Hex5dHex3HexNAc4NeuAc1 + 2Na
2435.794	2435.7863	-3.2	Hex5dHex1HexNAc4NeuAc2 + 3Na
2633.9146	2633.8993	-5.8	Hex6dHex2HexNAc6 + 1Na

Table S1. Summary of identified N-glycans detected by MALDI MSI of PNGase F-treated tissue.

Identified Protein	Protein Score	Identified Peptide + O-GlcNAc site	Peptide Score
Hemoglobin subunit alpha	1354	AVGHLDDLPGALS[+203.079]TLSDLHAHK	506
Hemoglobin subunit beta	1249	VHLS[+203.079]SEEK	501
		VHLSS[+203.079]EEK	437
Alcohol dehydrogenase 1	1843	FS[+203.079]LDPLITNVLPFEK	595
Pro-interleukin-16	560	PSALAT[+203.079]R	334
PDZ and LIM domain 5	823	EVVKPVPIT[+203.079]SAVSK	764
		EVVKPVPITSAVS[+203.079]K	582
Ornithine aminotransferase	909	T[+203.079]FQK	375
Host cell factor C1	815	SPITIIT[+203.079]TK	476
Lactoylglutathione lyase	745	SLDFYT[+203.079]R	354
Hemoglobin subunit gamma	1156	LLVVYPWT[+203.079]QR	375
Calnexin	1211	SKPDT[+203.079]STPPPSPK	595
		SKPDTSTPPPS[+203.079]PK	562
Histone H2B	894	GIMNS[+203.079]FVNDIFERIAGEASR	441
Aldehyde dehydrogenase 6	1195	EGAS[+203.079]ILLDGR	308

Table S2. Summary of identified O-GlcNAc-modified peptides with Byonic scores >300.

Theoretical *m/z*	Observed *m/z*	ppm error	Protein name	Peptide Sequence	PEP Score (PD)
1105.6000	1105.5990	-0.9	Vinculin	YAIGILNEGR	314.95
2546.2885	2546.2720	-6.5	Fumarate hydratase	THTQDAVPLTLGQEFSGYVQQVK	342.34
1274.6991	1274.6990	-0.1	Myosin heavy chain 9	YEILTPNSIPK	1641.10
1531.7136	1531.7000	-8.9	Hemoglobin alpha	IGSHGGEYGAEAVER	417.50
1786.8759	1786.8650	-6.1	Ribose-phosphate diphosphokinase	THNGESVSYLFSHVPL	335.00
2565.2440	2565.2600	6.2	Tryptophan-tRNA ligase	DMNQVLDAYENKKPFYLYTGR	272.02

Table S3. Summary of peptide identifications based on intact mass within 10 ppm and PEP scores >300 from LC-MS/MS data set matched to MSI data.

	OGA	PNGaseF	Trypsin	CHCA
Concentration (mg/mL)	0.35	0.1	0.01	10
Nozzle temp (°C)	30	45	30	75
# Passes	15	15	12	3
Flow rate (mL/min)	0.025	0.025	0.01	0.12
Track velocity (mm/min)	1200	1200	750	1200
Track spacing (mm)	3	3	3	3
Nozzle height (mm)	40	40	40	40
N_2 pressure (psi)	10	10	10	10
Track pattern (type)	CC	CC	HH	HH

Table S4. Summary of HTX M5 sprayer parameters used for each enzymatic reaction. The track pattern can be described as crisscross (CC) or horizonal-horizontal (HH) spray patterns.
